# Strategies to Address COVID-19 Vaccine Hesitancy and Mitigate Health Disparities in Minority Populations

**DOI:** 10.3389/fpubh.2021.645268

**Published:** 2021-04-23

**Authors:** Kate W. Strully, Teresa M. Harrison, Theresa A. Pardo, Jordan Carleo-Evangelist

**Affiliations:** ^1^Department of Sociology, University at Albany, State University of New York, Albany, NY, United States; ^2^Department of Communication, University at Albany, State University of New York, Albany, NY, United States; ^3^Center for Technology in Government, Public Administration and Policy, University at Albany, State University of New York, Albany, NY, United States; ^4^Office of Government and Community Relations, University at Albany, State University of New York, Albany, NY, United States

**Keywords:** vaccine hesitancy, COVID-19, health disparities, community-engaged campaigns, health equity

## Abstract

Beyond the complex logistical task of prioritizing, distributing and safely storing millions of doses of COVID-19 vaccines, state and local governments must simultaneously devise and carry out transparent plans that center equity and overcome the barriers to vaccination facing minority communities. Using insights gleaned from four focus groups conducted with health care and social service professionals serving minority communities in New York State as well as from existing research on vaccination, our results emphasize that vaccine hesitancy and access barriers—particularly within minority communities—pose significant hurdles to achieving widespread uptake of COVID-19 vaccines. Overcoming barriers requires community-engaged campaigns that acknowledge and address the historical injustices and on-going inequities that drive distrust within communities of color, emphasize understandable and culturally appropriate messages that directly address people's concerns about vaccine safety and access, and tap existing community infrastructure to make full use of trusted voices to deliver timely and accurate information about vaccines. Given emerging data and changing conditions, campaigns must also be self-reflective and adaptive, assessing progress and outcomes and reevaluating strategies as needed. However, above all, primary goals should remain focused on transparency, equity and building trust.

## Introduction

The COVID-19 pandemic has delivered many sobering lesson–among the most pressing the need to confront disparities in COVID-19 outcomes in minority communities^1^. Disproportionate rates of infection and death from COVID-19 within communities of color have laid bare the tragic consequences of systemic and pervasive inequities in medical care and working, living, and environmental conditions (1). While some states successfully contained early waves of COVID-19, the policies, institutions and cultural forces responsible for underlying systemic racial-ethnic inequities remain and threaten to find new expression as states carry out unprecedented mass-vaccination campaigns (2, 3). Beyond the enormously complex logistical task of distributing and safely storing millions of doses of vaccine, each state must simultaneously take steps to address vaccine hesitancy. Vaccine hesitancy impacts many demographic groups in America, but especially minority groups, whose experiences and viewpoints are shaped by legacies of past medical abuses, contemporary health care and criminal justice disparities, and unequal burdens of the COVID-19 pandemic.

The discussion below draws on existing research on vaccine hesitancy and disparities as well as on focus group comments from health care and social service professionals doing work related to community health serving minority communities in New York State. We focused on these professional groups as they have knowledge of communities' health behaviors and concerns, play key roles in vaccine delivery and information, and often serve as trusted sources for health information. The four focus groups, described in greater detail in Table 1, were conducted in August 2020 and organized around four locations: Long Island, Brooklyn/Queens, Syracuse, and Buffalo. Table 2 lists the primary questions related to vaccine concerns and barriers posed in the focus groups and key themes that arose in discussions. Taken together, existing research and these voices offer a compelling argument that efforts to address vaccine hesitancy must be multi-faceted, prioritize community engagement, and address the systemic inequities shaping people's experiences and viewpoints. This perspective article is based on a prior report made available in October 2020 (4), but includes updated recommendations to reflect new challenges facing equity goals in COVID-19 vaccination campaigns, and to contribute to the evidence base.

**Table 1:** Focus group format, recruitment, and participants.

**Focus group formats**	**All focus groups were conducted via Zoom, lasting ~90 min, during August 2020**
Recruitment	This convenience sample was recruited in partnership with Healthcare Association of New York State (HANYS). HANYS sent a broad notification about the study to members involved with community health work. Individuals indicating interest in participating were connected to the project team and provided details and Zoom invites. Some individuals who received the initial notification may have shared with others in their professional network, some of whom may have joined the group. Participants were asked to join groups according to where in NY State they practiced, and sites were selected to reflect multiple regions of the state • Long Island, a downstate largely suburban location, had 4 participants • Brooklyn/Queens, a downstate urban location, had 9 participants • Syracuse, an urban/suburban location in central upstate, had 7 participants • Buffalo, a primarily urban location in western upstate, had 11 participants
Participants' descriptions of their professional roles included:	Community health educator, community health worker, community health worker manager, civil rights organization board member, health care administration and management, health coordinator for early childhood education program, non-profit and/or social service administration, city development planner, non-profit and/or social service administration, nurse practitioner, nurse, nutritionist
Participants' descriptions of communities they work with referenced:	The following racial-ethnic-nativity groups: Caucasian, African American/African descent/Black, Latino/Hispanic (some specifying Spanish-speaking), Native American, Asian (including specific mention of Chinese, Korean, Bangladeshi, and Indian communities), Caribbean (including specific mention of Jamaican communities), Arab/Middle Eastern, immigrants, newly settled refugees Age ranges referenced included young children through seniors

**Table 2:** Primary questions and key themes of responses.

**Participants were asked to address two primary sets of questions. Listed below are the key themes that emerged from these group discussions**.	
**Question**	**Key themes**
**Concerns about vaccines:**	
What are some of the biggest concerns that members of your community have about a COVID-19 vaccine?	• Varied concerns related to safety and side effects, with particular emphasis on how the speed of vaccine development and political pressure could potentially compromise vaccine safety/efficacy (LI, B/Q, S, B) • Changing information, mixed messages, and misinformation about vaccines and virus itself driving distrust (LI, B/Q, S, B) • Justified distrust based on historical medical abuse (Tuskegee experiment, experimentation under slavery, as well as other cases) and on-going disparities (LI, B/Q, S, B) • Teenagers and young people not feeling as vulnerable to disease and may therefore be less motivated to take vaccine (LI, B/Q, S) • Black Lives Matter movement as an important context for understanding vaccine concerns (LI, S) • Cultural barriers between western medicine and some refugee communities leading to miscommunication and distrust (S) • Concerns about being among the first groups (either as essential workers or communities with high COVID-19 rates) when side effects will be least known (LI) • Vaccine trials may not include adequate representation of racial-ethnic groups or individuals with preexisting conditions (B)
What do you think are the best strategies for addressing concerns and making people feel more comfortable taking a COVID-19 vaccine?	Groups noted central importance of: • Linguistically and culturally appropriate messages delivered by trusted partners who believe in vaccine (B/Q, S, B) • Early engagement before vaccines are available and education along the way (LI, B/Q)
	Points on message content: • Acknowledging and having open and honest conversations about past abuses and ongoing disparities and making clear intentions and plans to do better (B/Q, S) • Attending to age/generational differences in message content (B/Q, B) • Clear information on what was done to speed up vaccine testing/production to address concerns about speed (B/Q)
	When asked to suggest trusted partners, participants noted: • Places of worship (LI, B/Q, B), Health care providers (LI, B/Q, S), Local community-based organizations (e.g., immigration assistance, domestic violence support (LI, B/Q, B), Service fraternities and sororities (LI, B), Young activists in communities (B/Q), Shelters (LI), Mutual aid networks (B/Q), Unions (B/Q), Sports leagues (B/Q), Higher ranking individuals within school system (S), County health officials (S)
	When discussing how to deliver messages, participants noted: • Ethnic news outlets and media, including social media (LI, B/Q, B) and radio (B/Q, LI)
**Access barriers to vaccination:**	
What are the biggest logistical and practical barriers that people are likely to face in getting the vaccine?	Transportation (LI, B/Q, S), Child care (LI, S), Time and scheduling around work (B/Q, S), Potential distribution sites that are unequally distributed across a city, participants noted this as a barrier with testing that might emerge with vaccine sites as well (B/Q)
What would be the best strategies for distributing a vaccine?	• Have messages in plain language that are accessible to persons with disabilities, and include messaging strategies that don't require internet (B)
	When discussing possible distribution sites, participants noted: • Chain drug stores (LI, B/Q, S, B), Places of worship (LI, B/Q, S), Schools (LI, B/Q, S), Health centers (B/Q), Community-based organizations (B/Q, B), Public libraries (B/Q, B), Shelters (B/Q), Public housing (B/Q), Parks (B/Q), and using mobile vaccine units (B) • Some participants (LI, S) voiced concerns that vaccines delivered in some sites or by some organizations might be perceived as linked to delivery of in-kind benefits or services, which could make some groups feel obligated to accept vaccinations, e.g., if vaccines are delivered at a site that also serves as a food bank, etc.

## Understanding Vaccine Hesitancy

Vaccine hesitancy refers to delays in the acceptance of or outright refusal of vaccines and is driven by attitudes toward a given vaccine, or the disease it is meant to prevent, as well as the difficulties one faces in trying to get the vaccine. A now-common phenomenon, vaccine hesitancy is context specific^2^, with reasons that vary by vaccines and groups (5, 6). Often motivated by feelings of disconfirmation by government and other elites to the felt needs and concerns of the public and perceived infringement of personal choice, vaccine hesitancy can also stem from cultural suspicions within some religious, ethnic and racial groups as illustrated by resistance to measles, mumps, and rubella (MMR) immunization within ultra-Orthodox Jewish communities in New York (7, 8). Black people and Hispanics in the United States also are more skeptical of MMR vaccine benefits and rate the risk of side effects higher than Whites (9). In Latin American immigrant communities, barriers to vaccine uptake include questions about the necessity of vaccinations, a lack of guidance by health care providers, a lack of insurance or access to health care, and concerns about side effects and safety (10). Within Black communities, vaccine hesitancy is associated with a longstanding and justified distrust of the medical community based on past experiments such as the Tuskegee syphilis study, as well as contemporary inequities in access to health care and in health outcomes (11). Underscoring the importance of this history, the Tuskegee experiments were spontaneously mentioned by participants in each of the focus groups as an enduringly salient legacy for black people. A participant in the Syracuse group working with newly resettled refugee communities also emphasized that large cultural barriers between western medicine and some refugee communities contribute to distrust and would likely pose barriers to vaccination.

Several national polls conducted in early-mid 2020 found approximately one-quarter to one-third of Whites and nearly half of black people and Hispanics appeared unwilling to be vaccinated against COVID-19 (12–15). However, more recent data indicate that public opinion is changing as vaccines are distributed. One survey reported that Black respondents who had gotten a vaccine or wanted to get one as soon as possible rose from 20% in December 2020 to 35% in January 2021; the number wishing to “wait and see” declined from 52 to 43%. Similarly, Hispanic respondents who reported getting a vaccine or wanting to get one as soon as possible rose from 26 to 42%; the number wishing to “wait and see” declined from 43 to 37% (16). Vaccine decision-making is an ongoing process, and it is critical to address recently documented disparities in Black and Hispanic Americans' access to quality information on how vaccines are developed and tested (17, 18).

Practical and logistical factors also present substantial hurdles to vaccination and drive hesitancy in minority communities–including lack of health insurance and/or access to a regular source of care, difficulty scheduling health care visits around employment or care-giving responsibilities, transportation, and language barriers. Several such barriers were noted by focus group participants (see Table 2). Some states have sought to reduce access barriers in their vaccine distribution so far, but in certain cases have run into unanticipated complications, highlighting how barriers can emerge at many points in access. As one telling example, New York state placed vaccination sites in minority neighborhoods hit hardest by COVID-19 to facilitate access for community members, but found that wealthy Whites from other areas were taking a large share of appointments, until the state began limiting early access to some sites to only local residents (19, 20).

## Centering Equity in Allocation Strategies Requires Building Trust

Several states and localities have integrated equity strategies in their vaccine distribution plans, including using community social disadvantage indicators in allocate vaccines more equitably (21, 22). However, the ability of these plans to achieve equity goals depends critically on early engagement and trust-building. Consider, for instance, examples from the 2009 H1N1 pandemic. H1N1 vaccination rates were characterized by racial-ethnic disparities roughly on par with those for seasonal flu vaccines, with the lowest coverage among African Americans (23). Researchers who studied the H1N1 campaign in Los Angeles described substantial challenges based on underlying distrust of government and community-generated informal messaging that framed H1N1 vaccines as a conspiracy to harm minority community members. In offering lessons learned from the Los Angeles campaign, they concluded, “The key to a successful emergency response relies on trust building and collaboration with community partners in the preparedness phase….” If public health authorities do not make “significant efforts to understand and address the issues of trust and disparities that exist at baseline,” they continued, “inequities will continue in the context of an emergency response” (24).

Our focus groups underscored these issues, highlighting how perceptions of a “rushed” vaccine interact with minority communities' experiences of historical and ongoing inequities in health care as well as contemporary movements for racial justice. Participants in each focus group agreed enthusiastically with the need to collaborate early and establish trust. As one participant from Brooklyn/Queens put it: “We have institutions that come into communities and kind of rain their benevolence down on the community instead of making the community a trusted, invested partner from the very beginning. … I think you get the community involved early, often and make them invested in the process and invested in the success.” Another participant from Long Island emphasized the importance of early, systematic education of key constituencies about the vaccine-development process: “[Don't] just come to me with, ‘Hey a vaccine and it's ready’, but educate me along the way so that I can have buy-in to it… Because if you wait until you get a vaccine, forget about it. They don't trust the process.”

## Addressing Vaccine Hesitancy Through Interventions

Our focus groups were conducted shortly after the large, early outbreak in New York City and during a period when media outlets regularly reported on controversial promises of vaccines by Election Day. When asked to describe concerns in the communities they serve, professionals in our focus groups most commonly cited efficacy, safety and trust, although some also mentioned complacency about the disease as a potential barrier for some subsets of teenagers or young adults. The European Union's Centers for Disease Prevention and Control (ECDC) emphasizes the importance of transparency when designing interventions to address the root causes of hesitancy and highlights three general areas where transparency should be improved: (1) basic information about how vaccines work, (2) the procedures for testing and licensing, as well as efficacy and side effects, and (3) conflicts of interest/trust in motives. These three areas align well with the responses from our focus groups (6).

### Basic Information

Some people hesitate to vaccinate because they misunderstand how vaccines work. This could be addressed with more basic information about vaccines presented at various education levels to accommodate varying levels of literacy. The current situation with COVID-19 is rife with seemingly contradictory information about what is necessary to produce a safe and effective vaccine, particularly with respect to the speed of vaccine development. The resulting confusion was mentioned in all four focus groups and is reflected in the comments of one focus group participant from Long Island: “We're getting saturated and inundated with information. Misinformation, correct information, who knows what kind of information. You don't know what is what. So you hear that it takes 18 months to do a vaccine. And now you're hearing that, poof, a vaccine can be done in less than that and … it's like, who's gonna get to the finish line first? I'm not sure if the one who get[s] to the finish line first is going to be the most effective one because it should have taken 18 months….”

### Testing/Licensing and Efficacy/Side Effects

Clearly communicating the procedures for testing and licensing a COVID-19 vaccine will be particularly important given the media's focus on the speed of vaccine testing. Participants across all four groups emphasized the special need to create trust on the subject of testing, as indicated by this participant from Brooklyn/Queens: “There's a distrust about the vaccines and no matter what happens, or how much outreach is done, unless we can get a clear message across that the vaccines are tested, [that] they're worthwhile, it's not going to work. And this mad rush to get a vaccine in right away is not going to help.” Involving minority communities in vaccine trials and vaccine preparation work would help foster trust that the vaccine's effectiveness and potential side effects have been adequately studied. Transparency about efficacy and risk is challenging, but there are several relevant recommendations from the ECDC, including emphasizing (1) “there is no such thing as a ‘perfect’ vaccine which protects everyone who receives it AND is entirely safe for everyone,” (2) “Effective vaccines … may produce some undesirable side effects which are mostly mild and clear up quickly,” and (3) “It is not possible to predict every individual who might have a mild or serious reaction to a vaccine, although there are a few contraindications to some vaccines” (25).

### Conflicts of Interest/Motives/Trust

The ECDC emphasizes the need to communicate about concerns arising from the profit motives of pharmaceutical companies. In the context of U.S. race relations, there will need to be further culturally sensitive efforts to build trust in medicine and vaccines for minority, immigrant and disadvantaged communities that are often marginalized from services and have been subjected to historical medical abuse or mistreatment. This was underscored in strong terms by one of our focus group participants from Long Island: “We have such a long history in this country being experimented on unwillingly or without informed consent, and I know that we are all taught that and it's … almost embedded in our culture. We all know about Tuskegee, we all know what happened to the slaves. We're not taught that in school but taught that by our own. And so when you talk about vaccines and when you talk about getting them, someone always brings it up. And so there's an inherent mistrust.” The participant went on to describe having heard that Black students might be among the first to return to school and continued: “Why [are] we always the first one[s] that they want to experiment on? Then it goes back to, ‘Because our lives don't matter.’ And so with the Black Lives Matter movement … building momentum now and COVID hitting at the same time… I think the numbers willing to take the vaccine will even be lower than the polls say.” People want to know that sources they trust endorse the vaccine. These sources may be inside or outside of the healthcare system and could take the form of community health workers, educators, patient advocates, community leaders, pharmacists, neighbors, health departments and trusted voices on social media. Good information delivered by trusted sources empowers members of the public to advocate for themselves and their loved ones. A participant from Brooklyn/Queens said understanding and responding to the “historical narrative” will be essential to successful vaccination messages that resonate in Black and other minority communities: “When we talk about vaccination, the idea of being a guinea pig is quite important … This is how we have to approach vaccination, in particular the Black community, but I think there's also additional ripple effects into other minority groups as well.”

Despite the fact that generalized trust in society and institutions is lower in minority communities, most people, regardless of race or ethnicity, trust people they know and have a history with—an esteemed member of their community or their own doctor—more than government health authorities and pharmaceutical companies (26, 27). Participants across all four groups emphasized that community-engaged vaccination strategies for COVID-19 will be essential to change attitudes and reduce access barriers in minority communities and preventing further COVID-19 health disparities. As one participant from the Syracuse focus group put it, there needs to be “an honest open conversation that's held in communities across the state about the disparities in health care and acknowledging … what has gone wrong, what this moment has shown us has gone wrong.” This conversation, the Syracuse participant noted, is especially relevant amid the broader social reckoning highlighted by the Black Lives Matter movement. “It almost seems like, you know, the blame is placed on the … marginalized community for not believing in medicine, when in actuality it's been the medical industry that has mistreated them.”

## Discussion and Recommendations

In recent months, multiple COVID-19 vaccines have received emergency authorization in the U.S. and have begun to be distributed to groups defined in early phases of states' distribution plans. Despite this rapidly evolving situation, key themes that emerged in the focus groups still ring true. Justified distrust remains a central challenge, as does frequently changing information, now related to vaccine eligibility, supply, and efficacy against new strains. Although many states featured equity goals in their vaccine distribution plans (22), among the 27 states reporting racial-ethnic information in COVID-19 vaccination data, vaccination rates among Whites are three times higher than those for Latinx persons and twice as high as those for African Americans (28). These sobering numbers highlight the challenge of achieving equity, as well as why it is vital for state and local governments to regularly assess outcomes, diagnose problems, and correct course as needed.

At the time the focus groups were completed in August 2020, it was clear that above all else states' efforts to center equity in their COVID-19 vaccine strategies should begin as soon as possible, ideally well in advance of the vaccine distribution program, be multi-faceted and prioritize community engagement. Key recommendations our group made previously included: establishing vaccine equity task forces with diverse membership reflecting community leaders and public health representatives; capitalizing on existing community structures to foster community engagement; developing vaccine up-take campaigns that directly address communities' concerns and social justice contexts; and making use of diverse communication channels. Here we update several of our recommendations to reflect current conditions.

First, beyond establishing vaccine equity task forces, states and localities should integrate several practices that are likely to increase chances of success and lasting improvement in health disparities. **Task forces should define clear objectives and specific metrics** that can be used to assess progress and identify and address barriers when progress has been insufficient (29). Implementing this practice will require making available data on vaccinations that can be broken down by multiple dimensions of inequity, including race-ethnicity, socioeconomic status, and geography, among others. States and localities should work to **maximize communication and collaboration between equity task forces and other related task forces**, such as those working on vaccine distribution and implementation, so equity goals can be centered through all parts of vaccine campaigns. This may be particularly important if states and localities must quickly react to contingencies to avoid vaccine spoilage. Without careful planning, the urgency inherent in these contingencies may pose barriers to achieving equity. Additionally, and importantly, states and localities should build on COVID-19 related task forces and the bright light that the current pandemic has shone on systemic racism and health disparities to **develop sustainable infrastructure for health equity and justice**. Michigan has been highlighted as a promising example on this point as their gubernatorial administration has used current momentum around COVID-19 disparities to convene a Black Leadership Advisory Council to advise the governor long-term on legislation that perpetuates race inequality and support for Black arts and community groups, and a Poverty Task Force, tasked with helping to develop an anti-poverty agenda for the state (30, 31).

Second, beyond engaging with trusted community organizations and leaders, **states and localities should support community organizations with needed funds and resources** so they can expand their work to address vaccine equity. The importance of community engagement and building on existing community structures has been widely recognized at both the federal and state/local levels (22, 32), however it is also important not to lose sight of the heavy burden that many community organizations are already shouldering as they support communities facing multiple hardships of the pandemic, including food insecurity, unemployment and of course the disease itself. Particularly in our Brooklyn/Queens focus group, participants shared that their organizations were already stretched so thin addressing their communities' increased needs during the pandemic that supporting vaccine efforts could pose a significant challenge. Michigan has again been cited as a promising example on this point as their vaccine equity task force has solicited applications to rapidly fund promising initiatives from community organizations (29).

Third, vaccine up-take campaigns already face significant hurdles as they attempt to address communities' concerns about vaccine safety and efficacy in culturally appropriate ways that address social justice contexts. The vaccine shortages and shifting eligibility criteria that characterize the current state of COVID-19 vaccination in the U.S. only make building that trust more challenging, particularly in communities that have traditionally been marginalized from services. While supply and distribution conditions are rapidly shifting and increased vaccine supply may soon alleviate some of this pressure, **states and localities should nonetheless build on existing partnerships and messaging strategies to provide culturally sensitive information about vaccine supply and eligibility as well**.

## Data Availability Statement

The data is not available for sharing to protect participants' identities.

## Ethics Statement

The studies involving human participants were reviewed and approved by University at Albany, SUNY, Institutional Review Board. Written informed consent for participation was not required for this study in accordance with the national legislation and the institutional requirements.

## Author Contributions

KS, TH, and TP contributed to the development of the protocol and conducted the focus groups. KS and TH were primarily responsible for the review of the literature. All authors contributed to the writing of the paper.

## Conflict of Interest

The authors declare that the research was conducted in the absence of any commercial or financial relationships that could be construed as a potential conflict of interest.

## Footnotes

^1^While we recognize that the terms used by members of different communities to refer to themselves vary, to simplify comparisons of COVID-19-related data to existing U.S. Census data, we use the term Black in this article in place of the full U.S. Census category “Black or African American alone”, and we use the term Hispanic to refer to the full Census category “Hispanic or Latino.” For the same reason, we use the term White to refer to individuals identified in the Census as “White alone, not Hispanic or Latino.”

^2^Many of the reasons for vaccine hesitancy discussed here focus on the U.S. social and historical context. There may be some parallels in other nations and some of the recommendations that follow may apply to other national contexts as well. Cross-national comparisons may shed additional light on vaccine hesitancy, however such comparisons are beyond the scope of this piece.
